# Economic costs of chemotherapy-induced febrile neutropenia among patients with non-Hodgkin’s lymphoma in European and Australian clinical practice

**DOI:** 10.1186/1471-2407-12-362

**Published:** 2012-08-22

**Authors:** Derek Weycker, Aurelie Danel, Anne Marciniak, Kate Bendall, Michael Lipsitz, Ruth Pettengell

**Affiliations:** 1 Policy Analysis Inc. (PAI), 4 Davis Court, Brookline, MA, 02445, USA; 2 Amgen Europe, Dammastrasse 23, Zug, 6300, Switzerland; 3 Amgen Ltd, 1 Uxbridge Business Park, Sanderson Road, Uxbridge, UB8 1DH, UK; 4 Cascade, 3rd Floor, 3 Copthall Avenue, London, EC2R 7BH, UK; 5 Department of Haematology, St. George’s University of London, Cranmer Terrace, London, SW17 0RE, UK

**Keywords:** Febrile neutropenia, Costs and cost analysis, Non-Hodgkin’s lymphoma

## Abstract

**Background:**

Economic implications of chemotherapy-induced febrile neutropenia (FN) in European and Australian clinical practice are largely unknown.

**Methods:**

Data were obtained from a European (97%) and Australian (3%) observational study of patients with non-Hodgkin’s lymphoma (NHL) receiving CHOP (±rituximab) chemotherapy. For each patient, each cycle of chemotherapy within the course, and each occurrence of FN within cycles, was identified. Patients developing FN in a given cycle (“FN patients”), starting with the first, were matched to those who did not develop FN in that cycle (“comparison patients”), irrespective of subsequent FN events. FN-related healthcare costs (£2010) were tallied for the initial FN event as well as follow-on care and FN events in subsequent cycles.

**Results:**

Mean total cost was £5776 (95%CI £4928-£6713) higher for FN patients (n = 295) versus comparison patients, comprising £4051 (£3633-£4485) for the initial event and a difference of £1725 (£978-£2498) in subsequent cycles. Among FN patients requiring inpatient care (76% of all FN patients), mean total cost was higher by £7259 (£6327-£8205), comprising £5281 (£4810-£5774) for the initial hospitalization and a difference of £1978 (£1262-£2801) in subsequent cycles.

**Conclusions:**

Cost of chemotherapy-induced FN among NHL patients in European and Australian clinical practice is substantial; a sizable percentage is attributable to follow-on care and subsequent FN events.

## Background

Neutropenia is a common side effect of myelosuppressive chemotherapy. Neutropenia both increases the risk of infection and diminishes patients’ ability to fight infection. Since fever is a cardinal sign of infection, when neutropenic patients develop fever (i.e., febrile neutropenia [FN]), the high likelihood of infection and serious consequences usually necessitates hospitalization for urgent evaluation, ongoing monitoring, and administration of intravenous antibiotics. FN, as well as severe or prolonged neutropenia, can lead to dose-delays, dose-reductions, and/or chemotherapy discontinuations, interfering with the delivery of optimal treatment and possibly adversely affecting patient outcomes [1-5].

The economic cost of chemotherapy-induced FN is substantial [6-8]. In one study, the mean cost of FN-related hospitalizations in the US was reported to be $8100 for patients with solid tumors, $11,600 for patients with non-Hodgkin’s lymphoma (NHL), and $28,000 for patients with leukemia [7]. It is likely, however, that this study--as well as other studies utilizing a cross-sectional design and hospital records--underestimated the total economic burden of FN, since the cost of follow-on care (e.g., post-discharge outpatient encounters) and subsequent FN events that may be related to the (initial) FN event were not considered. In a more recent US study employing a matched cohort design and considering all such costs, the economic burden of the initial FN event was estimated to represent only about 60% of the total cost of FN-related care, suggesting that prior studies may have underestimated the mean cost of FN-related events by as much as 40% [9].

Relatively little is known about the economic burden of chemotherapy-induced FN in European and Australian clinical practice, however, and no studies conducted outside the US have considered the burden of care for the initial FN event as well as the downstream consequences of the initial event. The current analysis, therefore, was undertaken to evaluate the full economic impact of FN among NHL patients receiving chemotherapy with cyclophosphamide, doxorubicin, vincristine, and prednisolone every 14 or 21 days (i.e., CHOP-14 or CHOP-21) in geographically diverse centers across Europe and in Australia.

## Materials and methods

### Study setting and source population

Data for this study were obtained from a large combined retrospective/prospective observational evaluation of supportive care among NHL patients receiving CHOP-14 or CHOP-21 chemotherapy in geographically diverse centers primarily across Western and Southern Europe (97% of patients) and in Australia (3% of patients). The design of this study, termed the “IMPACT NHL Study” (ClinicalTrials.gov: NCT00903812), is described in detail elsewhere in the literature [10-13]. In countries where it was required, ethical approval of the IMPACT NHL Study was obtained and patients provided written informed consent.

Briefly, approximately 1800 adult patients with NHL who were planned to receive at least three cycles of CHOP-14 or CHOP-21 chemotherapy--with or without rituximab--were recruited (about 600 retrospectively, 1200 prospectively) from 128 geographically diverse study centers. Study patients may have received a prior course of chemotherapy (~9% of study population). Patients enrolled retrospectively included those who completed all cycles of chemotherapy--regardless of actual number of cycles or outcomes thereof--after January 1, 2005 and prior to site participation in the study. Patients enrolled prospectively included all patients for whom CHOP-14 or CHOP-21 chemotherapy was planned for administration between January 2006 and December 2008; no additional interventions were required as a result of participation in the study. All patients enrolled in the IMPACT NHL Study (n = 1,864) who met protocol-defined eligibility criteria and received at least one cycle of chemotherapy (n = 1,829) were included in the source population for the current analysis.

For each patient, baseline data were collected on patient demographics (e.g., age, sex, race, height, weight), medical history (e.g., comorbid conditions), Eastern Cooperative Oncology Group Performance Status (ECOG PS), cancer characteristics (e.g., bulky disease, bone marrow involvement), International Prognostic Index (IPI) score (non-follicular patients only)/Follicular Lymphoma International Prognostic Index (FLIPI) score (follicular patients only), prior antineoplastic therapy, planned chemotherapy (i.e., CHOP-14 or CHOP-21, with or without rituximab [“-R”], doses, number of cycles), and predicted risk of FN (i.e., <20% vs ≥20%). Cycle-specific data collected during the chemotherapy course included actual chemotherapy regimen (i.e., drug, dose, date of administration), use of selected drugs/services (e.g., granulocyte colony-stimulating factor [G-CSF] for prophylaxis [primary or secondary] or treatment, erythropoiesis-stimulating agent [ESA], transfusions, anti-infective agents), performance status, and occurrence of FN--as well as selected other adverse outcomes--and associated setting of care (i.e., hospital, outpatient setting, home). Hospitalizations for reasons other than FN were also captured.

### Study population

The study population, consisting of patients who experienced FN and matched comparison patients, was selected from the source population as follows. For each patient in the source population, all chemotherapy cycles in which FN occurred were identified. FN was defined as a single oral temperature of ≥38.3^0^C or a temperature of ≥38.0°C for ≥1 hour, and a neutrophil count of <0.5 x 10^9^/L or a neutrophil count of <1.0 x 10^9^/L that is predicted to fall below 0.5 x 10^9^/L. Patients with FN in more than one cycle were classified according to the cycle number in which FN first occurred.

Patients in the source population who developed FN during their first cycle of chemotherapy (“FN patients”) were matched on selected covariates--hypothesized to be possibly associated with FN and healthcare costs--to patients who did not develop FN during their first cycle of chemotherapy (“comparison patients”), whether or not they developed FN in any subsequent cycles. Matched cycle-one FN patients and comparison patients were then removed from their respective pools. From remaining patients in the source population (i.e., those not previously matched), those who first developed FN in their second cycle of chemotherapy were matched to those who did not develop FN in that cycle. Matched cycle-two FN patients and comparison patients were then removed from their respective pools. FN patients and comparison patients were similarly matched in the third and all subsequent cycles. The cycle in which patients were matched was designated the “index cycle”.

Matching was implemented--sequentially, on a cycle-specific basis--for each FN patient by identifying all candidate comparison patients matching that FN patient in terms of age (±5 years), tumor stage, and chemotherapy regimen. The candidate with the closest propensity score (“nearest neighbor”) to the FN patient was selected as the matched comparison patient. Matching was conducted for FN patients randomly, and without replacement of comparison patients (i.e., comparison patients were matched to one FN patient only). Propensity scores were estimated using multivariate logistic regression; independent variables included all demographic and disease-specific characteristics (e.g., age, sex, IPI/FLIPI score, bone marrow involvement), predicted risk of FN, presence of (current/continuing) comorbidities, chemotherapy regimen, supportive care, ECOG PS, absolute neutrophil count (ANC), and presence of anemia symptoms (e.g., fatigue/tiredness, pallor/pale skin, headache, dyspnea). All of the above-listed variables--with the exception of ANC, presence of anemia symptoms, and supportive care--were characterised using data collected at baseline in the IMPACT NHL Study; the exceptions were characterised using data collected during the pre-index cycles and index cycle, as appropriate.

An alternative, more restrictive matching procedure--including age (±5 years), country of residence, tumor stage, bone marrow involvement, IPI/FLIPI score, and chemotherapy regimen when identifying candidate comparison patients--was first employed, but was ultimately deemed by study investigators to be inadequate due to small sample size (n = 118). The characteristics of FN patients and comparison patients matched using this more restrictive procedure is described in Additional file 1: Table S1 of the online supplement.

### Study measures

FN-related healthcare utilization was tallied for each FN patient and matched comparison patient from the cycle in which FN first occurred (for the former) through the last cycle of chemotherapy. Healthcare utilisation was examined in terms of: the number of FN events requiring inpatient care, outpatient care, home care, and other/unknown care, respectively; the total number of hospital days for all FN-related admissions that occurred from the index cycle through the end of the last chemotherapy cycle; and use--and reason for use--of G-CSF (pegfilgrastim, filgrastim, and other G-CSF [presumably lenograstim]) and IV antimicrobial agents by setting of care. Use of G-CSF and antimicrobial agents as prophylaxis was considered in post-index cycles only, while use of these agents as treatment was considered beginning with the index cycle.

FN-related healthcare costs were calculated for FN patients and comparison patients by combining estimates of FN-related healthcare utilization--as described above--with unit-cost data from the United Kingdom (UK). UK-specific unit costs were estimated from the perspective of the National Health Service using data from readily-available secondary sources and published literature, where available, as well as expert opinion, where needed. Principal sources of cost data were the 2009–2010 National Health Service (NHS) Schedule of Reference Costs (hospital costs), the 2010 Unit Costs of Health and Social Care Report from the Personal Social Services Research Unit (outpatient, home care, and other setting costs), and the British National Formulary (BNF), 60th Edition (pharmacotherapy costs) [14]. Unit costs are set forth in Table 1; a full description of methods employed to estimate unit costs is provided in Additional file 1: Table S2 of the online supplement.

**Table 1:** UK-specific unit costs of FN-related care

**Healthcare Resource**			**Unit Cost (£)**	**Source**
FN Inpatient Care				
	Long-Stay (per admission)		5361	NHS Reference Costs, 2008–2009 and 2009-2010
		Excess Bed Days (per day)	502	
	Short-Stay		772	
	Day Case		577	
FN Outpatient/Home/Other/Unknown Care				
	Outpatient Encounter			
		Initial Consultation	177	NHS Reference Costs, 2009-2010
		Follow-Up Consultation	116	
	Home Encounter		84	
	Other/Unknown Encounter		26	
	Drug*			
		Filgrastm (per 600 mcg)	59	BNF, 60th Edition
		Pegflgrastim (per 6 mg)	686	
		Other CSF (per day)	63	
		IV Antimicrobial Agents (per day)	38	

*Units and corresponding costs based on recommended dosing of agents.

### Statistical analyses

The adequacy of the matching procedure was evaluated based on the similarity of matched FN and comparison patients in terms of their baseline characteristics. Categorical variables were compared using the McNemar or Bowker test, and continuous variables were compared using the paired-samples t-test. The similarity of matched patients and unmatched patients--in terms of their characteristics--also was examined.

FN-related healthcare utilization and costs from the index cycle through the end of the last cycle of chemotherapy were examined on a cumulative basis for each patient in the FN and comparison groups. Mean values were estimated for the two groups, as well as for differences between groups, on an overall basis during the index cycle and during post-index cycles. Hospital days for FN patients and comparison patients with missing data on admission/discharge dates were imputed based on corresponding subgroup-specific mean values. FN-related healthcare costs also were evaluated among subgroups of patients defined on the basis of key characteristics, including index cycle, chemotherapy regimen, and predicted FN risk. Ninety-five percent confidence intervals (95% CIs) were generated using techniques of nonparametric bootstrapping.

The appropriateness of combining FN-related healthcare utilization data from different countries with unit cost information from the UK was evaluated based on a comparison of country-specific mean numbers of patients developing FN by setting of care, and for patients requiring inpatient care, country-specific mean numbers of hospital days. These analyses were based on data from FN patients and comparison patients who were matched on the basis of the criteria set forth above (i.e., age, tumor stage, chemotherapy regimen, and propensity score) as well as country of residence. Results were summarized using means and 95% CIs; formal statistical tests for heterogeneity between patients in different countries were not undertaken due to small country-specific sample sizes.

## Results

### Patient characteristics

Among the 1829 NHL patients in the source population, 331 (18%) experienced one or more FN events--a total of 479 FN events--during their course of chemotherapy; the number of events, by cycle, occurred as follows: 1st cycle, 128 (27%); 2nd cycle, 64 (13%); 3rd cycle, 67 (14%); 4th cycle, 68 (14%); and 5th or later cycle, 152 (32%). The majority of FN events (77%) required inpatient care.

 A total of 295 FN patients were successfully matched (1:1)--on age (±5 years), tumor stage, and chemotherapy regimen, as well as closest propensity score--to comparison patients (Table 2). (Selected covariates are presented in Table 2; a full listing of covariates is provided in Additional file 1: Table S3 of the online supplement.) Mean (±SD) age was 63 (±12) years, 69% had stage III or IV disease, and 91% received CHOP-14-R (28%) or CHOP-21-R (63%). No covariate differed significantly between FN patients and comparison patients. Most patients were matched in cycle 1 (43%) or cycle 2 (14%), and the mean number of cycles from the index cycle through the end of chemotherapy was 4.5 (±2.1) for FN patients and 4.6 (±2.1) for comparison patients. Ninety-seven percent of the study population received chemotherapy at a site in Western or Southern Europe; the remaining 3% received care in Australia.

**Table 2:** Characteristics of FN patients and comparison patients matched on basis of age (±5 years), tumor stage, chemotherapy regimen, and propensity score

					**FN Patients (n = 295)**	**Comparison Patients (n = 295)**	P-value*
Demographics							
	Age, mean±SD, y				63.3 ± 12.4	63.0 ±12.1	0.108
	Male, %				49.0	50.7	0.674
	Country of Residence						
		Australia			2.7	2.7	0.817
		Austria			5.8	9.2	
		Belgium			11.6	11.9	
		France			24.5	18.4	
		Germany			10.2	12.2	
		Greece			2.7	1.7	
		Italy	5.1	7.8			
		Netherlands			8.8	8.5	
		Nordics			7.1	6.5	
		Portugal			1.4	1.7	
		Spain			12.6	12.6	
		Switzerland			2.7	2.4	
		UK and Ireland			4.8	4.4	
NHL							
	Tumor Stage						
	I				14.6	14.6	---
	II				16.7	16.7	
	III				19.4	19.4	
	IV				49.3	49.3	
	IPI/FLIPI Score						
		IPI Score					
			Low		16.4	20.8	0.368
			Intermediate		50.4	50.6	
			High		19.6	17.3	
			Missing		13.6	11.4	
		FLIPI score					
			Low Risk		11.4	15.4	0.920
			Intermediate Risk		36.4	23.1	
			Poor Risk		43.2	51.3	
			Missing		9.1	10.3	
		Bone Marrow Involvement			29.6	24.8	0.252
Clinical							
	Risk of FN, %						
		<20%			27.0	27.0	0.564
		≥ 20%			73.0	73.0	
	Medical Conditions, %						
		Cardiovascular Disease			23.5	25.2	0.615
		Respiratory Disease			7.8	4.8	0.128
		Gastrointestinal Disease			6.1	5.1	0.564
		Renal Disease			3.1	3.4	0.796
		Hepatic/Biliary Disease			2.7	5.8	0.061
		Haematologic/Lymphatic			5.1	3.1	0.221
		Immunologic			5.4	3.7	0.317
		Other			49.0	46.3	0.505
	ECOG PS, %						
		0			47.6	52.4	0.182
		1			31.0	31.6	
		2			10.2	7.5	
		3			5.1	3.7	
		4			1.0	0.7	
		Missing			5.1	4.1	
	Hematology						
		ANC (X10^^^9L), mean±SD			5.4 ± 4.5	5.7 ± 3.4	0.794
		Presence of Anemia Symptoms, %			56.8	56.1	0.856
Treatment							
	Chemotherapy Regimen, %						
		CHOP-14			3.40	3.40	---
		CHOP-14-R			27.89	27.89	
		CHOP-21			5.44	5.44	
		CHOP-21-R			63.27	63.27	
	Supportive Care, %						
		Index Cycle					
			G-CSF Prophylaxis		51.7	56.5	0.162
			Anti-Infective FN Prophylaxis		13.3	11.6	0.529
		Pre-Index Cycle					
			G-CSF				
				Prophylaxis	37.4	41.8	0.074
				Treatment	9.5	6.5	0.128
			Anti-Infective				
				FN Prophylaxis	7.8	5.8	0.303
				FN Treatment	1.7	0.3	0.102
			Transfusion		9.5	6.8	0.144
	Index Cycle Number*						
	1				42.9	42.9	---
	2				13.6	13.6	
	3				11.2	11.2	
	4				12.2	12.2	
	5				9.9	9.9	
	6				8.2	8.2	
	7				1.7	1.7	
	8				0.3	0.3	
	Total Number of Cycles (incl. index cycle thru end of course)						
	1				10.6	11.2	0.812
	2				11.9	9.5	
	3				13.3	11.6	
	4				12.3	15.3	
	5				12.6	11.6	
	6				24.2	24.8	
	7				4.1	4.1	
	8				10.9	11.9	

*P-values were not calculated for variables used in the matching process and for which there are no differences between groups (i.e., there was an exact match).

Matched and unmatched patients were similar in terms of many of their baseline characteristics, but matched patients were older (by about 4 years, on average) and a higher percentage of them had advanced disease (58% vs 45%) and a predicted risk of FN ≥20% (73% vs 61%). These results were not unexpected, since age and presence of advanced disease are risk factors for FN, and comparison patients were matched to FN patients based on these factors. (Data on all baseline characteristics for matched and unmatched patients are available in Additional file 1: Table S4 of the online supplement.)

### FN-related healthcare utilization

FN patients averaged 1.44 (95%CI 1.34-1.56) FN events from their index cycle through the end of their course of chemotherapy--0.44 (0.34-0.56) during post-index cycles--and comparison patients averaged 0.15 (0.10-0.21) such events over the same period, corresponding to a difference of 1.29 FN events (1.17-1.43) (Table 3). Among FN patients, 76% of index FN events and 79% of post-index FN events required inpatient care; for comparison patients, the corresponding percentage (during post-index cycles) was 84%. Among FN patients requiring inpatient care, the average number of FN events (irrespective of setting of care) was 1.10 (1.00-1.21) versus 0.13 (0.08-0.18) for matched comparison patients, a difference of 0.98 (0.85-1.11). Mean number of FN-related hospital days was 6.21 (5.28-7.17) for FN patients versus 0.63 (0.30-1.02) for comparison patients, a difference of 5.62 (4.64-6.62).

**Table 3:** Healthcare utilization among FN patients and matched comparison patients

				**Mean (95% CI)**								
				Overall*			**Index Cycle**			**Post-Index Cycle**		
**Healthcare Resources**				**FN Patients (n = 295)**	**Comparison Patients (n = 295)**	**Difference**	**FN Patients (n = 295)**	**Comparison Patients (n = 295)**	**Difference**	**FN Patients (n = 295)**	**Comparison Patients (n = 295)**	**Difference**
No. of FN Events				1.44(1.34, 1.56)	0.15(0.10, 0.21)	1.29(1.17, 1.43)	1.00(1.00, 1.00)	0.00(0.00, 0.00)	1.00(1.00, 1.00)	0.44(0.34, 0.56)	0.15(0.10, 0.21)	0.29(0.17, 0.43)
	Requiring Inpatient Care			1.10(1.00, 1.21)	0.13(0.08, 0.18)	0.98(0.85, 1.11)	0.76(0.71, 0.80)	0.00(0.00, 0.00)	0.75(0.70, 0.81)	0.35(0.26, 0.45)	0.13(0.08, 0.18)	0.22(0.12, 0.34)
	Requiring Outpatient Care			0.07(0.04, 0.11)	0.01(0.00, 0.03)	0.06(0.02, 0.09)	0.06(0.03, 0.09)	0.00(0.00, 0.00)	0.06(0.03, 0.09)	0.01(0.00, 0.03)	0.01(0.00, 0.03)	0.00(−0.02, 0.02)
	Requiring Home Care			0.18(0.12, 0.26)	0.01(0.00, 0.02)	0.17(0.10, 0.24)	0.12(0.08, 0.16)	0.00(0.00, 0.00)	0.12(0.08, 0.16)	0.06(0.02, 0.11)	0.01(0.00, 0.02)	0.05(0.01, 0.10)
	Requiring Other Care/No Action			0.05(0.02, 0.08)	0.00(0.00, 0.00)	0.05(0.02, 0.08)	0.04(0.02, 0.06)	0.00(0.00, 0.00)	0.04(0.02, 0.06)	0.01(0.00, 0.02)	0.00(0.00, 0.00)	0.01(0.00, 0.02)
	Requiring Unknown Care			0.04(0.02, 0.08)	0.00(0.00, 0.00)	0.04(0.02, 0.08)	0.03(0.01, 0.08)	0.00(0.00, 0.00)	0.03(0.01, 0.05)	0.01(0.00, 0.03)	0.00(0.00, 0.00)	0.01(0.00, 0.03)
Use of Healthcare Services												
	Inpatient Setting											
		Admissions		1.10(1.00, 1.21)	0.13(0.08, 0.18)	0.98(0.85, 1.11)	0.76(0.71, 0.80)	0.00(0.00, 0.00)	0.75(0.70, 0.81)	0.35(0.26, 0.45)	0.13(0.08, 0.18)	0.22(0.12, 0.34)
		Days in Hospital		6.21(5.28, 7.17)	0.63(0.30, 1.02)	5.62(4.64, 6.62)	4.13(3.56, 4.78)	0.00(0.00, 0.00)	4.15(3.57, 4.81)	2.08(1.40, 2.75)	0.63(0.30, 1.02)	1.48(0.74, 2.23)
		G-CSF (# admin.)		0.51(0.31, 0.75)	0.05(0.01, 0.10)	0.46(0.26, 0.69)	0.37(0.22, 0.53)	0.00(0.00, 0.00)	0.37(0.22, 0.53)	0.14(0.03, 0.27)	0.05(0.01, 0.10)	0.10(−0.03, 0.26)
			Filgrastim	0.33(0.18, 0.51)	0.03(0.00, 0.08)	0.30(0.15, 0.49)	0.23(0.13, 0.34)	0.00(0.00, 0.00)	0.23(0.13, 0.33)	0.10(0.01, 0.23)	0.03(0.00, 0.08)	0.07(−0.03, 0.22)
			Pegfilgrastim	0.00(0.00, 0.01)	0.00(0.00, 0.01)	0.00(−0.01, 0.01)	0.00(0.00, 0.01)	0.00(0.00, 0.00)	0.00(0.00, 0.01)	0.00(0.00, 0.00)	0.00(0.00, 0.01)	0.00(−0.01, 0.00)
			Other Agent	0.18(0.06, 0.33)	0.01(0.00, 0.04)	0.16(0.04, 0.31)	0.14(0.04, 0.27)	0.00(0.00, 0.00)	0.14(0.04, 0.25)	0.04(0.00, 0.11)	0.01(0.00, 0.04)	0.03(−0.03, 0.10)
		IV Antimicrobials (# admin.)		4.46(2.68, 7.51)	0.42(0.20, 0.68)	3.98(2.26, 6.89)	2.04(1.64, 2.47)	0.00(0.00, 0.00)	2.05(1.64, 2.48)	2.42(0.83, 5.25)	0.42(0.20, 0.68)	1.94(0.37, 4.70)
	Outpatient Setting											
		G-CSF (# admin.)		0.27(0.11, 0.47)	0.33(0.10, 0.62)	−0.06 (−0.27, 0.13)	0.03(0.00, 0.06)	0.00(0.00, 0.00)	0.03(0.00, 0.06)	0.24(0.08, 0.43)	0.33(0.10, 0.62)	−0.09(−0.30, 0.10)
			Filgrastim	0.19(0.05, 0.37)	0.24(0.02, 0.50)	−0.05 (−0.25, 0.12)	0.02(0.00, 0.05)	0.00(0.00, 0.00)	0.02(0.00, 0.04)	0.17(0.04, 0.36)	0.24(0.02, 0.50)	−0.07(−0.27, 0.11)
			Pegfilgrastim	0.08(0.03, 0.13)	0.09(0.03, 0.17)	−0.01(−0.10, 0.08)	0.01(0.00, 0.03)	0.00(0.00, 0.00)	0.01(0.00, 0.03)	0.06(0.02, 0.12)	0.09(0.03, 0.17)	−0.02(−0.11, 0.66)
			Other Agent	0.00(0.00, 0.01)	0.00(0.00, 0.00)	0.00(0.00, 0.01)	0.00(0.00, 0.01)	0.00(0.00, 0.00)	0.00(0.00, 0.01)	0.00(0.00, 0.00)	0.00(0.00, 0.00)	0.00(0.00, 0.00)
		IV Antimicrobials (# admin.)		0.00(0.00, 0.00)	0.02(0.00, 0.07)	−0.02(−0.07, 0.00)	0.00(0.00, 0.00)	0.00(0.00, 0.00)	0.00(0.00, 0.00)	0.00(0.00, 0.00)	0.02(0.00, 0.07)	−0.02(−0.07, 0.00)
	Home Setting											
		G-CSF (# admin.)		6.29(5.09, 7.54)	5.13(4.08, 6.28)	1.17(−0.30, 2.66)	0.13(0.04, 0.22)	0.01(0.00, 0.04)	0.11(0.03, 0.21)	6.16(4.99, 7.41)	5.12(4.07, 6.26)	1.06(−0.39, 2.52)
			Filgrastim	2.35(1.60, 3.24)	2.20(1.46, 3.01)	0.16(−0.89, 1.24)	0.07(0.02, 0.14)	0.00(0.00, 0.03)	0.06(0.00, 0.13)	2.28(1.53, 3.18)	2.19(1.46, 2.99)	0.10(−0.95, 1.17)
			Pegfilgrastim	1.07(0.88, 1.29)	0.96(0.77, 1.17)	0.12(−0.18, 0.38)	0.01(0.00, 0.02)	0.00(0.00, 0.01)	0.00(−0.01, 0.02)	1.07(0.88, 1.28)	0.96(0.77, 1.17)	0.11(−0.18, 0.38)
			Other Agent	2.86(1.92, 3.97)	1.97(1.19, 2.83)	0.90(−0.21, 2.03)	0.05(0.00, 0.11)	0.00(0.00, 0.00)	0.05(0.00, 0.11)	2.81(1.89, 3.92)	1.97(1.19, 2.83)	0.85(−0.25, 1.98)
		IV Antimicrobials (# admin.)		1.05(0.68, 1.47)	0.03(0.00, 0.09)	1.01(0.62, 1.45)	0.60(0.37, 0.87)	0.00(0.00, 0.00)	0.60(0.37, 0.86)	0.45(0.17, 0.78)	0.03(0.00, 0.09)	0.41(0.14, 0.74)
	Other/Unknown Setting											
		G-CSF (# admin.)		0.69(0.41, 1.02)	0.87(0.31, 1.57)	−0.18(−0.96, 0.47)	0.00(0.00, 0.01)	0.00(0.00, 0.00)	0.00(0.00, 0.01)	0.69(0.40, 1.02)	0.87(0.31, 1.57)	−0.19(−0.97, 0.47)
			Filgrastim	0.45(0.18, 0.77)	0.66(0.15, 1.33)	−0.23(0.94, 0.39)	0.00(0.00, 0.01)	0.00(0.00, 0.00)	0.00(0.00, 0.01)	0.45(0.18, 0.76)	0.66(0.15, 1.33)	−0.23(0.94, 0.39)
			Pegfilgrastim	0.23(0.14, 0.34)	0.15(0.07, 0.23)	0.09(−0.04, 0.22)	0.00(0.00, 0.00)	0.00(0.00, 0.00)	0.00(0.00, 0.00)	0.23(0.14, 0.34)	0.15(0.07, 0.23)	0.09(−0.04, 0.22)
			Other Agent	0.01(0.00, 0.04)	0.06(0.00, 0.17)	−0.04(−0.16, 0.03)	0.00(0.00, 0.00)	0.00(0.00, 0.00)	0.00(0.00, 0.00)	0.01(0.00, 0.04)	0.06(0.00, 0.17)	−0.04(−0.16, 0.03)
		IV Antimicrobials (# admin.)		0.00(0.00, 0.00)	0.00(0.00, 0.00)	0.00(0.00, 0.00)	0.00(0.00, 0.00)	0.00(0.00, 0.00)	0.00(0.00, 0.00)	0.00(0.00, 0.00)	0.00(0.00, 0.00)	0.00(0.00, 0.00)

*Mean levels of resource use from index cycle through end of the chemotherapy course.

Levels of FN-related healthcare utilization among FN patients and comparison patients (matched on the basis of the criteria set forth above plus country of residence [n = 236]) and corresponding differences between these groups, were largely comparable across countries (data available in Additional file 1: Table S5 of the online supplement). Among country-specific subgroups including ≥15 patients, mean number of FN events among FN patients ranged from 1.41-1.63, and mean number of events requiring inpatient care, 0.79-1.50; the mean number of hospital days among FN patients ranged from 3.8-5.2.

### FN-related healthcare costs

Mean overall FN-related healthcare cost among FN patients was £8066 (95%CI £7277-£8882)--£4051 (£3633-£4485) during the index cycle and £4015 (£3374-£4724) during post-index cycles (Table 4). Mean cost of FN care among comparison patients (for whom all costs were incurred during post-index cycles, by design) was £2290 (£1923-£2655). Overall mean cost thus was £5776 (£4928-£6713) higher for FN patients than comparison patients, with 71% of the difference attributable to care in the index cycle (£4051 [£3633-£4485]) and 29% attributable to care in post-index cycles (£1725 [£978-£2498]).

**Table 4:** Healthcare costs among FN patients and matched comparison patients

**Healthcare Resources**	**Mean (95% CI), in British pounds**								
	Overall*			**Index Cycle**			**Post-Index Cycles**		
	**FN Patients (n = 295)**	**Comparison Patients (n = 295)**	**Difference**	**FN Patients (n = 295)**	**Comparison Patients (n = 295)**	**Difference**	**FN Patients (n = 295)**	**Comparison Patients (n = 295)**	**Difference**
Total Cost of FN, Overall	8,066(7,277, 8,882)	2,290(1,923, 2,655)	5,776(4,928, 6,713)	4,051(3,633, 4,485)	---	4,051(3,633, 4,485)	4,015(3,374, 4,724)	2,290(1,923, 2,655)	1,725(978, 2,498)
Inpatient Care	6,007(5,278, 6,771)	543(287, 858)	5,463(4,709, 6,322)	3,942(3,520, 4,372)	---	3,942(3,520, 4,372)	2,065(1,478, 2,694)	543(287, 858)	1,522(900, 2,180)
Outpatient Care	180(123, 252)	130(56, 219)	50(−37, 144)	65(43, 89)	---	65(43, 89)	115(62, 182)	130(56, 219)	−15 (−97, 74)
Home Care	1,673(1,452, 1,899)	1,447(1,220, 1,674)	225(−78, 545)	41(26, 59)	---	41(26, 59)	1,632(1,409, 1,853)	1,447(1,220, 1,674)	184(−117, 503)
Other/Unknown Care	207(135, 293)	169(97, 253)	38(−67, 152)	4(2, 5)	---	4(2, 5)	203(131, 289)	169(97, 253)	34(−71, 148)
Total Cost of FN, by Setting of Care									
Inpatient Episode	9,688(8,709, 10,653)	2,430(1,984, 2,931)	7,259(6,327, 8,205)	5,281(4,810, 5,774)	---	5,281(4,810, 5,774)	4,407(3,613, 5,225)	2,430(1,984, 2,931)	1,978(1,262, 2,801)
Outpatient Episode	3,761(1,908, 6,007)	1,777(1,116, 2,565)	1,984(−193, 4,177)	341(293, 435)	---	341(293, 435)	3,420(1,529, 5,676)	1,777(1,116, 2,565)	1,644(−521, 3,853)
Home Episode	3,291(2,445, 4,339)	1,826(993, 2,919)	1,466(234, 2,495)	324(241, 415)	---	324(241, 415)	2,967(2,124, 4,059)	1,826(993, 2,919)	1,141(−99, 2,176)
Other/Unknown Episode	2,211(1,140, 3,425)	1,850(766, 3,157)	361(−956, 1,650)	104(51, 205)	---	104(51, 205)	2,107(1,052, 3,333)	1,850(766, 3,157)	258(−1,031, 1,558)

*Mean costs from index cycle through end of the chemotherapy course.

Stratified by the setting of care for the index FN event, the difference in mean overall cost was greatest for those who received inpatient care for FN during the index cycle versus comparison patients who were matched to them--£9688 (£8709-£10,653) versus £2430 (£1984-£2931), a difference of £7259 (£6327-£8205). Corresponding results for those patients whose index FN event was treated in other settings of care were: outpatient, £3761 (£1908-£6007) versus £1777 (£1116-£2565); and home care, £3291 (£2445-£4339) versus £1826 (£993-£2919).

Differences in mean FN-related healthcare costs between FN patients and comparison patients were comparable within subgroups defined on the basis of key characteristics, including index cycle, chemotherapy regimen, and predicted FN risk. By index cycle, differences were: cycle 1, £6024 (£4588-£7429); cycle 2, £4913 (£3123-£6875); cycle 3, £6029 (£3409-£9242); cycle 4, £6804 (£4519-£9543); and cycle ≥5, £5121 (£4084-£6220). For patients receiving CHOP-14-R and CHOP-21-R, differences were £5667 (£4002-£7261) and £6045 (£4855-£7173), respectively. For matched FN and comparison patients with a predicted FN risk ≥20%, difference was £5603 (£4603-£6605), and for those with a predicted FN risk <20%, £6006 (£4557-£7783).

## Discussion

Using a matched-cohort design, healthcare utilisation data for 1829 NHL patients receiving CHOP-14 or CHOP-21 chemotherapy in European and Australian clinical practice, and UK-specific unit costs, we estimated the total economic impact of FN including care for the initial event as well as downstream FN-related care. The mean economic burden of FN (i.e., the difference in costs between FN patients and comparison patients) was found to be substantial, totaling £5776 per patient. For FN patients whose index event required hospitalization, economic burden totaled £7259 (US$11,610), which is comparable to the estimate (US$12,397) from the study by Weycker et al. (2008) that employed the same study design and data from a large US healthcare claims database (2001–2003) [9]. Most of the total economic burden was attributable to FN inpatient admissions, since 76% of index events and 79% of post-index events required hospitalization, and the (unit) cost of such care is considerably higher than that for FN treated in the outpatient or home care settings. As in the study by Weycker and colleagues, while the burden of the initial event was substantial and accounted for the majority of FN-attributable costs (70%), a significant minority of these costs resulted from follow-on care and subsequent events underscoring the economic importance of accounting for the downstream consequences of this complication. The economic costs of index events averaged £4051, versus £1725 for FN-related healthcare utilization in post-index cycles.

For several reasons, our estimates of the total economic burden of FN may be conservative. First, for FN events requiring hospitalization, follow-on care that may have occurred in the outpatient setting subsequent to hospital discharge--in the index cycle or subsequently--was not collected during the IMPACT NHL study and thus could not be incorporated into our estimates of disease burden. Second, for all FN events--irrespective of care setting--additional resources that may have been used (e.g., laboratory supplies), and additional services that may have been provided (e.g., drug administration), in the treatment of FN--and that are not included in assumed unit costs/reimbursed values--could not be incorporated due to data limitations. Finally, we erred on the conservative side when making assumptions about the frequency and intensity of resource use outside the hospital setting--where such data were not available from the IMPACT NHL Study--and thus may have underestimated corresponding costs of care. We also note that estimates of disease burden were robust when employing the alternative (i.e., more stringent) matching criteria for FN patients and comparison patients, when admissions designated as “elective” were excluded from estimation of (unit) cost of hospital care, and when using Payment by Results (PbR) tariffs (rather than NHS reference costs) for hospital care (data available in Additional file 1: Tables S6-S8 of the online supplement).

Several limitations of our study are noteworthy. First, although FN patients and comparison patients were matched on several characteristics, it is possible that the two cohorts differed in terms of unobserved characteristics that predispose them to FN. To the extent that FN patients in our study population were more likely to develop FN than comparison patients, some downstream costs--in particular, those occurring in cycles after the one in which the initial (i.e., index) FN event occurred--may not be attributable to the initial FN event *per se.* Especially problematic in this regard are the costs of subsequent FN events. To what extent does experiencing a first episode of FN increase a patient’s risk of subsequent episodes, relative to the extent to which the risk of subsequent episodes is predicted by the same risk factors associated with the initial episode? If largely the latter, the actual burden of chemotherapy-induced FN may be closer to the total costs of the index event plus any additional follow-on care that is directly related to the initial event--most of which, presumably, would occur in the same cycle. Second, it is possible that certain biases in recording may exist such that patients who experience an FN event may be more likely to have FN noted on future encounters versus patients without a history of these complications, all else equal, which could upwardly bias our estimates. Third, although FN-related healthcare utilization appears to be comparable across countries, differences between the UK and other countries in the services or intensity of services that are provided within a given setting (and that are not captured in the study database) are not reflected in study results. Accordingly, caution should be exercised in generalizing the results of this study to other settings. Fourth, we note that FN patients comprise those whose index course was their first course of chemotherapy as well as those who previously received chemotherapy. To the extent that the consequences of FN are different based on prior receipt of chemotherapy, results may not be generalizable to these two subgroups (i.e., subgroups comprising solely patients who previously received chemotherapy and those who did not, respectively). Fifth, we note that various patient- and provider-specific factors may influence the pattern and intensity of FN treatment, on an inpatient and outpatient basis, and that to the extent these factors vary across settings, study results may not be fully generalizable to other patient populations. Sixth, the IMPACT NHL Study was not designed for economic analyses, and thus data on certain types of healthcare utilization (e.g., outpatient visits for follow-on care) were not collected, while other data (e.g., identification and use of IV antimicrobial agents as treatment or prophylaxis) may not always have been collected or classified consistently and comprehensively. Therefore, the results of this analysis may underestimate the total attributable cost of FN. Finally, while our study population comprised adult patients with NHL, we note that the Healthcare Resource Group version 4 (HRG4) code--PA45Z--that was used to cost FN-related inpatient care is grouped in the chapter “Diseases of Childhood and Neonates”. We also note, however, that this code has been employed in several single technology appraisals (STAs) and evidence review group’s (ERG) reports to cost chemotherapy-related febrile neutropenia among adults with HER2-negative metastatic breast cancer, adults with chronic lymphocytic leukemia, adults with metastatic prostate cancer, and adults with squamous cell carcinoma of the head and neck, respectively [15-18].

## Conclusion

In conclusion, the economic burden of FN among NHL patients in European and Australian clinical practice is substantial, and a significant proportion of this burden is due to the downstream consequences of the condition. Studies focusing only on the initial FN event may underestimate the total attributable cost of these complications.

## Authors’ contributions

Authorship was designated based on the guidelines promulgated by the International Committee of Medical Journal Editors (2004). All persons who meet criteria for authorship are listed as authors on the title page. The contribution of each of these individuals to this study--by task--is as follows: conception and supervision (Weycker, Danel), development of design (Weycker, Danel, Marciniak, Bendall), conduct of analyses (Weycker, Lipsitz), interpretation of results (all authors), preparation of manuscript (Weycker, Lipsitz), and review of manuscript (all authors). All authors have read and approved the final version of the manuscript.

## Declaration of funding

Funding for this research was provided by Amgen Europe. The study sponsor reviewed and approved the study research plan and study manuscript; data management, processing, and analyses were conducted by PAI, and all final analytic decisions were made by study authors.

## Declaration of competing interests

Derek Weycker and Michael Lipsitz are employed by PAI (Brookline, MA, USA). Aurelie Danel is employed by Amgen Europe (Zug, Switzerland). Anne Marciniak was employed by Amgen Ltd. (Uxbridge, UK) at the time the study was conducted. Kate Bendall is a consultant and funded by Amgen Ltd. (Uxbridge, UK). Ruth Pettengell is employed by St. George’s University of London, Department of Haemotology (London, UK), and was a principal investigator for the IMPACT Study.

## Financial support

Funding for this research was provided by Amgen Europe GmbH to Policy Analysis Inc. (PAI).

## Pre-publication history

The pre-publication history for this paper can be accessed here:

http://www.biomedcentral.com/1471-2407/12/362/prepub

## Supplementary Material

Additional file 1 **Table S1.** Characteristics of FN and comparison patients matched on basis of original criteria in sSAP; **Table S2.** UK-specific unit costs of FN-related care; **Table S3.** Characteristics of FN patients and comparison patients matched on basis of age (±5 years), tumor stage, chemotherapy regimen, and propensity score; **Table S4.** Characteristics of full patient population, unmatched patients, and matched patients; **Table S5.** Healthcare utilization among FN patients and matched comparison patients matched, by country; **Table S6.** Healthcare costs among FN patients and comparison patients matched on basis of age (±5 years), country of residence, tumor stage, bone marrow involvement, IPI/FLIPI score, and chemotherapy regimen; **Table S7.** Healthcare costs among FN patients and matched comparison patients, excluding hospital admissions designated as “elective” from calculation of unit costs; **Table S8.** Healthcare costs among FN patients and matched comparison patients, using PbR tariffs as basis of unit cost of hospital care.(XLS 469 kb) Click here for file
